# Prevalence of degenerative signs of the temporomandibular joint in the Canarian population through the analysis of panoramic radiographs: A pilot study

**DOI:** 10.4317/jced.63004

**Published:** 2025-09-01

**Authors:** Marilia Betancor-Pérez, Rocío Trinidad Velázquez-Cayón, Juan Francisco Loro-Ferrer, María Fernanda Cortés-Sylvester

**Affiliations:** 1 Doctorate Program in Health Sciences, Faculty of Health Science, University of Las Palmas de Gran Canaria (ULPGC), Las Palmas de Gran Canaria, Spain; 2 Department of Dentistry, Faculty of Health Sciences, University Fernando Pessoa Canarias; 3 Centro universitario San Isidoro, Pablo de Olavide University, Seville; 4 Clinical Sciences Department, Faculty of Health Science, Las Palmas de Gran Canaria University (ULPGC), Las Palmas de Gran Canaria, Spain; 5 Department of Dentistry, Finis Terrae University, Santiago de Chile, Chile

## Abstract

**Background:**

Degenerative diseases of the temporomandibular joint (TMJ) represent a frequent subtype of temporomandibular disorders (TMDs), particularly prevalent among individuals over the age of 40. Although cone-beam computed tomography (CBCT) is recognized as the diagnostic gold standard for evaluating bony alterations of the TMJ, panoramic radiography remains widely utilized in clinical practice due to its accessibility and lower radiation exposure.

**Material and Methods:**

A retrospective observational pilot study was conducted at the Dental Clinic of the University Fernando Pessoa Canarias. A simple random sample of 60 panoramic radiographs from patients over 40 years of age was selected from a database comprising 323 records collected between April and October 2024. Bilateral assessment of the TMJs was performed by two independent observers according to established diagnostic criteria for degenerative alterations. Inter-observer agreement was measured using Cohen’s Kappa statistics. Associations between degenerative and indeterminate findings and demographic variables were evaluated using the Chi-square (χ²) test. Descriptive statistics for age were reported as means and interquartile ranges.

**Results:**

Among the 120 TMJs evaluated, osteophyte formation was the only degenerative finding detected (30%). Condylar flattening, considered an indeterminate sign, was observed in 85% of cases. Osteophytes were more frequently identified in males (33.33%); however, no statistically significant association was found between osteophyte presence and sex (*p* = 0.655). A significant association was observed between the presence of osteophytes and condylar flattening (χ² = 4.73, *p* = 0.030). Inter-observer agreement was moderate for degenerative signs (κ = 0.68) and minimal for indeterminate signs (κ = 0.37).

**Conclusions:**

Osteophyte formation was the sole radiographic indicator of TMJ degeneration identified in this sample, frequently co-occurring with condylar flattening. Although panoramic radiography serves as a valuable screening modality, its diagnostic limitations underscore the need for CBCT in cases with suspected or advanced degenerative joint changes.

** Key words:**Temporomandibular joint, Osteoarthritis, Degenerative joint disease, Panoramic radiography, Osteophyte.

## Introduction

Temporomandibular disorders (TMDs) comprise a complex group of musculoskeletal conditions and represent the second most common cause of orofacial pain [1,2], as well as the second most prevalent musculoskeletal condition overall [3]. The reported prevalence of TMDs is variable, as clinical manifestations do not always correlate with radiographic findings [4]. These disorders most frequently manifest between the ages of 20 and 40 [2,5], although age of onset may vary depending on the specific subtype.

Degenerative diseases of the temporomandibular joint (TMJ), with an estimated prevalence in the general population ranging from 8% to 60% [6,7], are more commonly observed in adulthood, with peak incidence typically occurring after the age of 50 [5]. Such conditions are also more prevalent in women, with a reported female-to-male ratio exceeding 2:1 [8,9].

According to the Research Diagnostic Criteria for Temporomandibular Disorders (RDC/TMD), 2014 [10], degenerative diseases of the TMJ include both osteoarthritis and osteoarthrosis. Although these terms are frequently used interchangeably in the literature [11,12], they are defined by distinct diagnostic criteria. Osteoarthritis is characterized by the degradation of articular cartilage accompanied by remodeling of the underlying subchondral bone tissues [3]. It is associated with low-grade chronic inflammation involving cartilage, subchondral bone, and synovial fluid, and is clinically manifested by pain, restricted mandibular movement at rest and during function, joint crepitus, and muscle weakness [13].

As the condition progresses and evolves chronically into a non-inflammatory phase [11], it may result in reduced joint space, marked remodeling of articular surfaces, persistent crepitus, and absence of pain—features that define osteoarthrosis [10,14]. In both conditions, clinical signs and symptoms have limited diagnostic validity and are not consistently present; therefore, radiographic assessment plays a critical role in the diagnosis, particularly for osteoarthrosis [14]. A confirmed diagnosis of degenerative TMJ disease requires the presence of at least one radiographic feature, such as osteophytes, erosion, subchondral cysts, or generalized sclerosis. In contrast, articular surface flattening and localized sclerosis are considered indeterminate signs, although they may represent early or adaptive changes [10].

Cone-beam computed tomography (CBCT) and conventional computed tomography (CT) are considered the gold standards for imaging evaluation of degenerative TMJ disease [5,10,15]. These modalities offer high spatial resolution for detecting osseous changes, but they are generally reserved for confirmatory diagnosis and require interpretation by trained professionals.

Panoramic radiography remains the most employed radiographic method in general dental practice for comprehensive assessment of dental structures and adjacent anatomical regions, owing to its low radiation dose, affordability, and ease of acquisition. In many dental settings, panoramic radiographs are routinely obtained—frequently even prior to clinical examination—for patients who meet basic age and health criteria [16]. While panoramic radiographs have recognized diagnostic limitations, they serve as a useful screening tool for the preliminary identification of TMJ alterations [17,18].

At the Dental Clinic of the University Fernando Pessoa Canarias, panoramic radiographs are routinely performed on all patients aged 16 and older during their initial visit, as part of the comprehensive diagnostic protocol. Consequently, radiographic findings involving the TMJ, particularly those suggestive of degenerative alterations, must not be overlooked, especially when considered alongside the patient’s medical history. In Spain, contemporary studies addressing the prevalence of radiographic signs of TMJ degeneration remain scarce [4].

Accordingly, the aim of this retrospective observational pilot study is to evaluate the prevalence of radiographic signs suggestive of degenerative TMJ disease in individuals over 40 years of age within the Canarian population, based on the analysis of panoramic radiographs.

## Material and Methods

A retrospective observational pilot study was conducted. Ethical approval was obtained from the Ethics Committee of the University Fernando Pessoa Canarias (approval report no. PI 004-2024).

- Sample Selection

To ensure unbiased selection, a simple random sampling method was applied to a database of 323 panoramic radiographs from patients aged over 40 who attended the dental clinic at the University Fernando Pessoa Canarias between April and October 2024. A total of 60 radiographs were randomly selected for inclusion in the study.

The selected panoramic radiographs were required to display both the right and left temporomandibular joints (TMJs). These radiographs had originally been acquired for general diagnostic purposes, regardless of the condition of the TMJs. Demographic data including patient age and biological sex were extracted from electronic medical records.

Radiographs were excluded if they met any of the following criteria: patients younger than 40 years of age; presence of artifacts that hindered accurate visualization and interpretation; evidence of TMJ prostheses or a history of TMJ surgery; or incomplete clinical data regarding age or biological gender.

- Image Interpretation

Two independent examiners assessed the selected images. The panoramic radiographs had been acquired using a Carestream CS 8100 panoramic unit (Carestream Health, Inc., USA) equipped with a Toshiba D-054 X-ray generator, operating at a tube voltage of 80kV, tube current of 8.0mA, and an exposure time of 10.8 seconds, yielding an absorbed radiation dose of 89.71mGy•cm². Images were stored in JPG format and analyzed using CS Imaging Software version 7.0.1.

Each examiner evaluated both the right and left condyles for the presence of degenerative radiographic signs, including generalized sclerosis (increased density of the cortical bone extending into the medullary bone), erosion (loss of continuity of the cortical bone and adjacent subcortical bone), subchondral cyst (well-defined osteolytic area confined to the subcortical bone), and osteophyte (marginal bony outgrowth on the condylar head). In addition, the presence or absence of indeterminate signs was assessed: condylar flattening (flattening of the bone contour deviating from its natural convex morphology) or localized sclerosis (increased bone density of the subcortical bone confined to a specific region) [10,15].

The relationship between indeterminate signs (flattening and localized sclerosis) and degenerative changes was also investigated. Each condyle was individually assessed for the presence or absence of these signs, and diagnoses were made separately for degenerative and indeterminate features.

- Statistical Analysis

An inter-observer agreement analysis was conducted to assess diagnostic consistency for both degenerative and indeterminate TMJ findings. Percentage agreement and Cohen’s Kappa coefficient (κ) were calculated using the irr package, version 0.84.1. This statistical approach provided a quantitative measure of diagnostic reliability between observers.

Interpreting the κ values followed McHugh’s classification [19]: 0-0.20 (none), 0.21-0.39 (minimal), 0.40-0.59 (weak), 0.60-0.79 (moderate), 0.80-0.90 (strong), and >0.90 (almost perfect agreement). Kappa values closer to 1.0 indicate high inter-observer consistency, whereas values near 0 suggest agreement by chance.

Chi-square (χ²) tests were applied to assess associations between categorical variables of interest. Descriptive statistics for patient age were reported as mean and interquartile range. A significance level of α = 0.05 was adopted for all analyses.

## Results

A total of 120 temporomandibular joints (TMJs) were evaluated. The sample included more women than men (72 vs. 48). The overall mean age was 57.09 ± 10.64 years (women: 57.5 ± 9.26; men: 56.48 ± 12.52). The findings recorded by each observer are presented in  Table 1.

- Degenerative Signs of the Temporomandibular Joint

**Figure 1:**
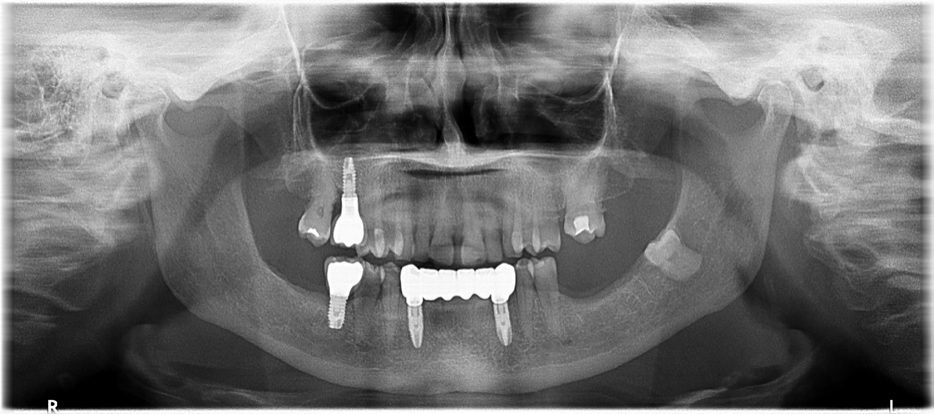
Panoramic radiograph showing osteophytes on the right and left condyles, along with condylar flattening in both temporomandibular joints.

Osteophyte formation was the only degenerative sign identified, with a prevalence of 30% (Fig. 1). Seventy percent of the evaluated TMJs showed no degenerative changes, and no additional degenerative signs—such as erosion, subchondral cysts, or generalized sclerosis—were detected.

Osteophytes were more frequently observed in men than in women (33.33% vs. 27.78%), although this difference was not statistically significant (χ² = 0.2, *p* = 0.655). The most prevalent age range among male patients was 57.69 years [44.93 – 75.44], and among female patients it was 59.66 years [49.77 – 62.35]. Osteophytes were more commonly found unilaterally (66.67%).

**Figure 2:**
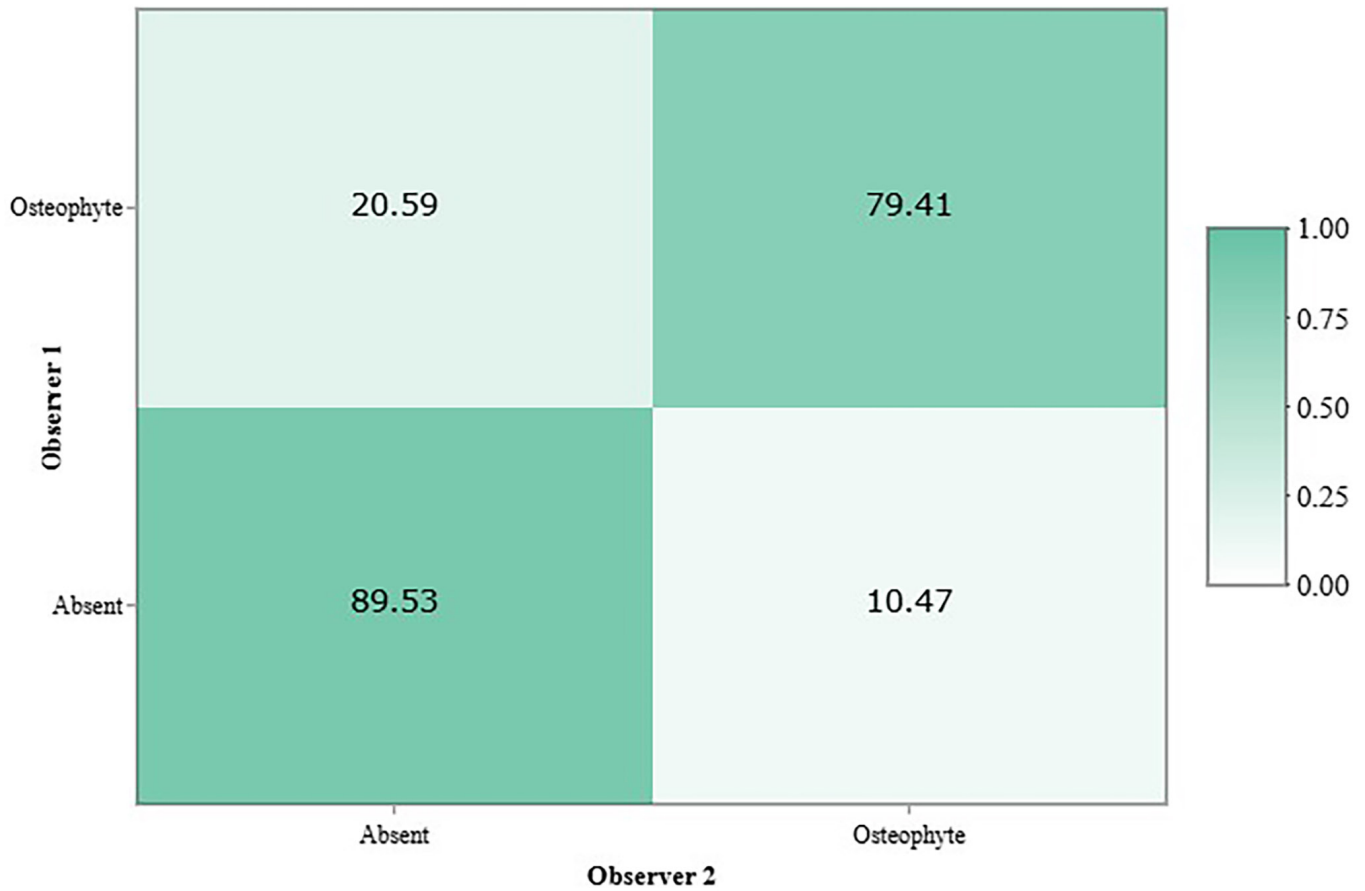
Inter-observer concordance matrix for the diagnosis of degenerative signs in the temporomandibular joint.

The inter-observer agreement for the diagnosis of degenerative TMJ signs was classified as moderate, with a Cohen’s Kappa coefficient of κ = 0.68 and an observed agreement rate of 86.67% (Fig. 2).

- Indeterminate Signs of the Temporomandibular Joint

The only indeterminate sign observed was condylar flattening, detected in 85% of the evaluated sample. This sign was more frequently identified in women (88.89%) than in men (79.17%), and it showed a tendency toward bilateral distribution (75.86%).

**Figure 3:**
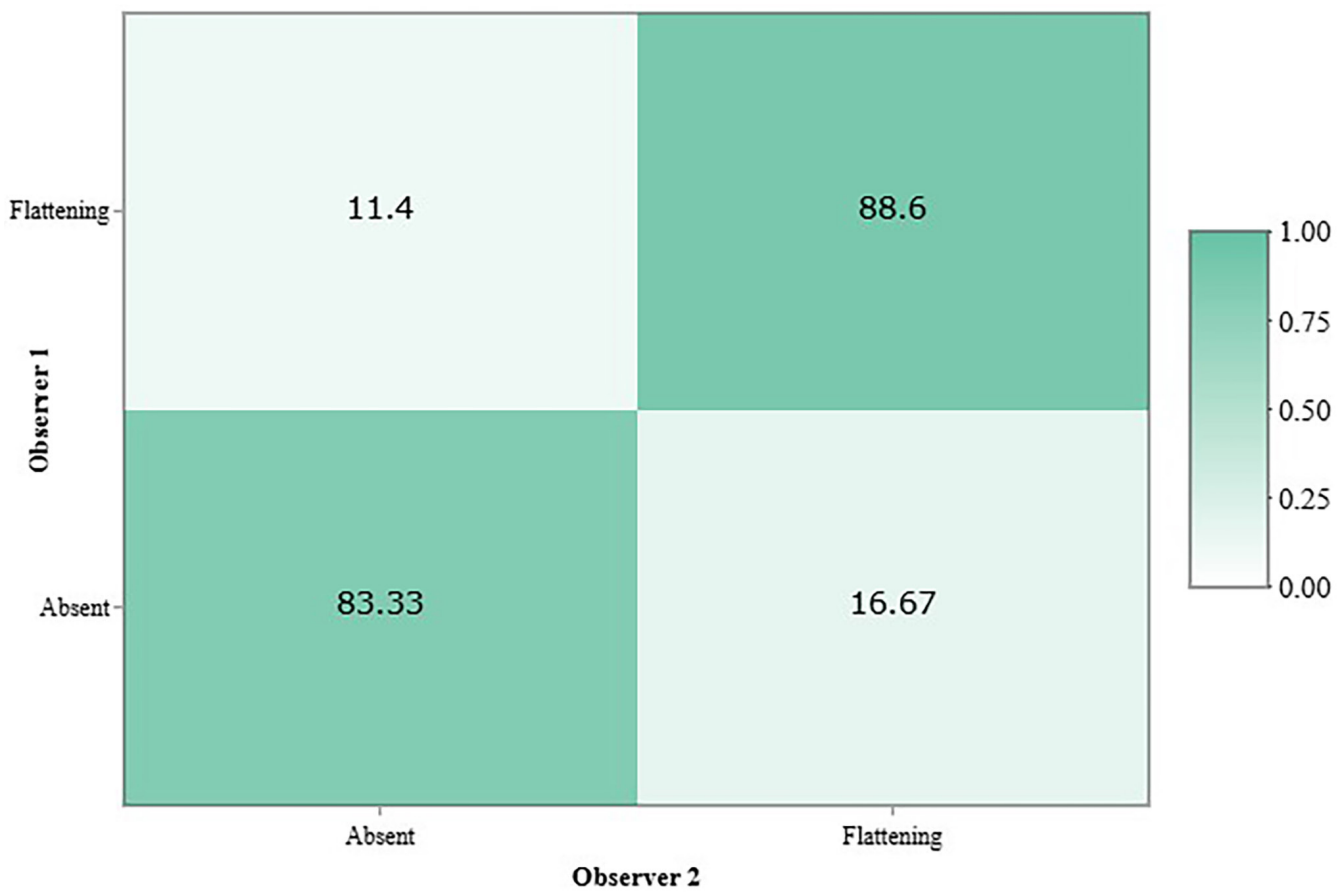
Inter-observer concordance matrix for the diagnosis of indeterminate signs in the temporomandibular joint.

The inter-observer agreement for indeterminate signs was minimal (κ = 0.37), despite a high overall agreement rate of 88.33%. The relatively low kappa value may be attributed to the limited number of cases without indeterminate findings; in such instances, a disagreement on a less frequent category (i.e., absence of flattening) weighs more heavily on the statistical index (Fig. 3).

- Correlation Between Indeterminate Signs and Degenerative Signs

**Figure 4:**
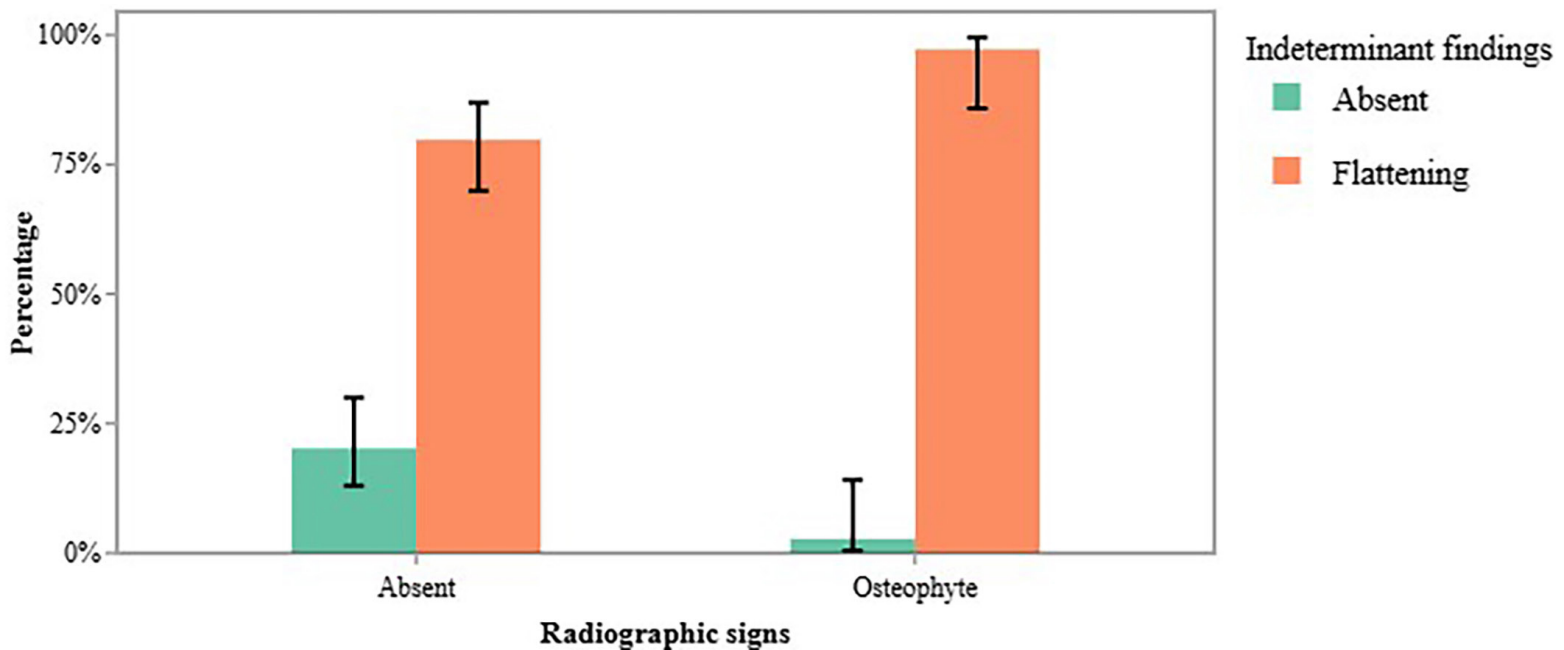
Association between osteophyte formation and condylar flattening in the temporomandibular joint.

Among the TMJs diagnosed with osteophytes, 97.22% also exhibited condylar flattening (Fig. 1), indicating a statistically significant association between these two findings (χ² = 4.73, *p* = 0.030) (Fig. 4).

## Discussion

The aim of this study was to evaluate the prevalence of degenerative signs in the condylar region of the temporomandibular joint (TMJ) using panoramic radiographs from a sample of the Spanish population. Considering that the average age of the population in Spain exceeds 40 years as of 2024 [20], 60 panoramic radiographs from patients over the age of 40 were selected, and the presence of degenerative and indeterminate signs at the condylar level was analyzed.

Panoramic radiography is a low-dose imaging modality that allows the assessment of multiple maxillofacial structures and is therefore widely utilized in dentistry as an initial diagnostic and screening tool for various disorders [21]. Although panoramic radiographs have inherent limitations, such as structural superimposition and reduced image sharpness [22,23], they have been shown to demonstrate high specificity in the detection of degenerative joint changes when such alterations are present [24]. For this reason, panoramic radiography should be considered a valid first-line imaging modality.

When comparing the sensitivity and specificity of panoramic radiography and magnetic resonance imaging (MRI) in the diagnosis of TMJ degenerative changes, both modalities exhibit excellent specificity but limited sensitivity, except for osteophyte detection by MRI, which is comparatively more sensitive [24].

Poveda-Roda *et al*. [25] found that 64.3% of patients clinically diagnosed with osteoarthritis exhibited degenerative signs on panoramic radiographs, while 90% showed such signs on MRI. Additionally, 57.1% of patients with a clinical diagnosis of osteoarthrosis demonstrated degenerative signs on panoramic radiographs, compared to 100% on MRI. Compared to cone-beam computed tomography (CBCT), however, panoramic radiographs provide lower image resolution and are less capable of detecting subtle degenerative changes, potentially leading to diagnostic errors or misinterpretations [26]. Consequently, panoramic radiography and MRI are more susceptible to false negatives when compared with CBCT, meaning that cases of joint degeneration may remain undetected when relying solely on these imaging techniques.

In this study, the inter-observer agreement for the diagnosis of degenerative signs was moderate (κ = 0.68), with an agreement rate of 86.67%. For indeterminate signs, agreement was minimal (κ = 0.37), despite an overall agreement rate of 88.33%. These levels of agreement are comparable to those reported in related studies.

Kaimal *et al*. [24], for example, compared the diagnostic accuracy of panoramic radiography and MRI with CBCT and found that inter-observer agreement was slight when panoramic radiographs were used (κ = 0.16; agreement: 19%), moderate with MRI (κ = 0.47; agreement: 59%,), and substantial with CBCT (κ = 0.71; agreement: 84%). Similarly, Ladeira *et al*. (26] observed inter-observer agreement values ranging from fair to moderate (0.22 ≤ κ ≤ 0.39) for panoramic radiographs, with intra-observer agreement ranging from slight to moderate (0.18 ≤ κ ≤ 0.45). Poveda-Roda *et al*. [25] reported intra-observer agreement at κ = 0.61. In contrast, agreement levels using CBCT were consistently higher, with both intra- and inter-observer κ values approaching excellence (κ = 0.75) [26].

The moderate inter-observer agreement for the detection of degenerative signs in the present study may be attributed to the fact that only osteophytes were identified, potentially facilitating their recognition due to reduced diagnostic ambiguity. On the other hand, the minimal agreement for indeterminate signs may be related to the high frequency of condylar flattening in the sample, possibly leading observers to overdiagnose this finding—even in its absence.

Osteophyte formation was the only degenerative sign identified (30%), and condylar flattening was the only indeterminate sign observed (85%). These findings are consistent with those of Poveda-Roda *et al*. [25], who reported that condylar flattening and osteophytes were the most frequently observed morphological changes in patients with degenerative TMJ conditions (mean age: 54.7 ± 20.2 years) when assessed using panoramic radiographs. When MRI was employed, erosion of the articular surfaces was also identified. Ladeira *et al*. [26] found that in patients aged between 18 and 75 years, the most prevalent morphological feature identified using both panoramic radiography and CBCT was condylar flattening (80%), followed by osteophytes (30%) and sclerosis (7%).

Conversely, CBCT imaging allows the identification of a broader range of degenerative changes. The most frequently detected sign on CBCT is erosion (34,35), followed by osteophytes [27-30], subchondral cysts [15,27,30], and generalized sclerosis [27,30]. Among the indeterminate signs, condylar flattening remains the most common, whereas localized sclerosis is less frequently observed [27-30].

In this study, the average age of patients presenting with degenerative signs was 57.69 years for men and 59.66 years for women. Previous studies have reported a higher prevalence of TMJ degenerative disease in women compared to men [15,27-29]. However, the findings of this study align with those reported by Pontual *et al*. [15], who observed the highest prevalence of TMJ degeneration in the 70–79 age group. Osteophyte formation was the most frequently reported degenerative sign (3%), followed by the combination of condylar flattening and osteophytes (29%). Condylar flattening was also the most prevalent indeterminate sign (59%). Similarly, in a study involving 65-year-old patients [27], condylar flattening remained the most identified indeterminate feature, while osteophytes were the most frequent degenerative sign, followed by erosion, subchondral cysts, and generalized sclerosis.

The collective findings of this study indicate that the prevalence of degenerative signs is associated with patient age [5]. In this regard, Alexiou *et al*. [28] analyzed TMJ degenerative pathology and demonstrated a statistically significant correlation between increasing age and the severity of degenerative alterations, with higher age associated with a greater prevalence of extensive erosion (*p* = 0.048) and extensive osteophyte formation (*p* = 0.003). The only degenerative signs identified in their study were erosion (59%) and osteophytes (55%).

Consistent with this observation, Dumbuya *et al*. [9) reported that in a population with a mean age of 73.35 ± 6.28 years, the most prevalent degenerative sign was subchondral cysts (63.3%), followed by osteophytes (60%) and erosion (17.8%). The most prevalent indeterminate sign was condylar flattening (37.8%), followed by localized sclerosis (24.4%).

In contrast, younger populations demonstrate a lower prevalence of subchondral cysts and localized sclerosis. For example, Kiliç *et al*. [29], in a study involving participants with a mean age of 30.75 ± 12.68 years, found an overall prevalence of subchondral cysts of 3.4%. Similarly, Derwich *et al*. [30], studying individuals with a mean age of 24.93 ± 7.74 years, identified subchondral cysts in 11% and generalized sclerosis in only 1% of cases.

Among degenerative signs, erosion is generally considered to be the earliest manifestation; however, its detection via panoramic radiography is rare unless the lesion exhibits significant depth and width. Other signs typically emerge in more advanced stages of disease progression. Due to its characteristic morphology and location, osteophyte formation is more easily visualized in panoramic images compared to other features that may be obscured by anatomical overlap or insufficient radiographic resolution [21].

Importantly, this study found a statistically significant correlation between the presence of osteophytes and condylar flattening (*p* = 0.030), with 97.22% of patients presenting with osteophytes also demonstrating condylar flattening. This relationship has also been documented in previous literature, where the combined presence of both findings was observed in 29% of cases [15]. In this study, osteophytes were predominantly unilateral (66.67%), whereas condylar flattening tended to be bilateral (75.86%), a distribution pattern also reported in prior research [27].

Based on the findings of the present study and corroborating evidence from prior studies, panoramic radiography (orthopantomography) cannot be considered a definitive or sufficiently reliable diagnostic tool for confirming degenerative TMJ disease. CBCT remains the recommended imaging modality for such diagnoses. Nevertheless, recognizing that CBCT is not routinely performed unless clinical signs of TMD are present, panoramic radiographs retain value as an initial screening tool, particularly in detecting cases with more advanced degenerative pathology (14). Given the prevalence rates observed in this study, degenerative changes detected on panoramic radiographs should not be disregarded during clinical evaluation.

In light of these findings, future studies will utilize CBCT as the radiographic modality of choice and will involve a larger, more representative sample encompassing the national population. It is important to emphasize that this pilot study provides preliminary data for a previously unexamined population and serves as a foundation for subsequent research incorporating more advanced diagnostic methods and rigorous methodologies to enhance scientific validity.

This pilot study is not without limitations. First, it did not include clinical variables that may be associated with the presence of TMJ degenerative disease. Second, the study sample was restricted to individuals over 40 years of age, thus excluding potentially informative data from younger populations. Third, the moderate and minimal inter-observer agreement indices observed may influence the reliability of the data. However, these values should be interpreted with caution, as the Cohen’s Kappa coefficient inherently penalizes disagreements more heavily when evaluating infrequent findings, while being less affected by disagreements on more prevalent signs due to their dilution in the total data set. Lastly, the small sample size and absence of CBCT as a comparative standard limit the generalizability of the findings and reduce their extrapolative power for broader populations.

In conclusion, the only degenerative sign identified in this retrospective prevalence pilot study conducted in the Canary Islands was the osteophyte (30%), which exhibited a strong correlation with condylar flattening. Thus, panoramic radiography revealed condylar degenerative changes of moderate prevalence in this population—findings that should not be overlooked during routine diagnostic assessments.

## Figures and Tables

**Table 1:** Agreement in the Diagnosis of Degenerative Temporomandibular Joint (TMJ) Disorders: Results by Observer and Demographic Subgroup.

Agreement in the diagnosis of degenerative TMJ disorders					
			Global	Male	Female
N			120	48	72
Age (x ± SD)			57.09 ± 10.64	56.48 ± 12.52	57.5 ± 9.26
Laterality	Right	% (CI95%)	50 % (41.19 -- 58.81 %)	50 % (36.39 -- 63.61 %)	50 % (38.75 -- 61.25 %)
Laterality	Left	% (CI95%)	50 % (41.19 -- 58.81 %)	50 % (36.39 -- 63.61 %)	50 % (38.75 -- 61.25 %)
Radiographic signs (Observer 1)	Absent	% (CI95%)	72.5 % (63.91 -- 79.7 %)	72.92 % (59 -- 83.43 %)	72.22 % (60.95 -- 81.24 %)
Radiographic signs (Observer 1)	Subchondral cyst	% (CI95%)	0 % (0 -- 3.1 %)	0 % (0 -- 7.41 %)	0 % (6.94e-16 -- 5.07 %)
Radiographic signs (Observer 1)	Erosion	% (CI95%)	0 % (0 -- 3.1 %)	0 % (0 -- 7.41 %)	0 % (6.94e-16 -- 5.07 %)
Radiographic signs (Observer 1)	Generalized sclerosis	% (CI95%)	0 % (0 -- 3.1 %)	0 % (0 -- 7.41 %)	0 % (6.94e-16 -- 5.07 %)
Radiographic signs (Observer 1)	Osteophyte	% (CI95%)	27.5 % (20.3 -- 36.09 %)	27.08 % (16.57 -- 41 %)	27.78 % (18.76 -- 39.05 %)
Indeterminant findings (Observer 1)	Absent	% (CI95%)	20 % (13.82 -- 28.04 %)	25 % (14.92 -- 38.78 %)	16.67 % (9.8 -- 26.91 %)
Indeterminant findings (Observer 1)	Flattening	% (CI95%)	80 % (71.96 -- 86.18 %)	75 % (61.22 -- 85.08 %)	83.33 % (73.09 -- 90.2 %)
Indeterminant findings (Observer 1)	Cortical sclerosis	% (CI95%)	0 % (0 -- 3.1 %)	0 % (0 -- 7.41 %)	0 % (6.94e-16 -- 5.07 %)
Radiographic signs (Observer 2)	Absent	% (CI95%)	70 % (61.28 -- 77.47 %)	66.67 % (52.54 -- 78.32 %)	72.22 % (60.95 -- 81.24 %)
Radiographic signs (Observer 2)	Subchondral cyst	% (CI95%)	0 % (0 -- 3.1 %)	0 % (0 -- 7.41 %)	0 % (6.94e-16 -- 5.07 %)
Radiographic signs (Observer 2)	Erosion	% (CI95%)	0 % (0 -- 3.1 %)	0 % (0 -- 7.41 %)	0 % (6.94e-16 -- 5.07 %)
Radiographic signs (Observer 2)	Generalized sclerosis	% (CI95%)	0 % (0 -- 3.1 %)	0 % (0 -- 7.41 %)	0 % (6.94e-16 -- 5.07 %)
Radiographic signs (Observer 2)	Osteophyte	% (CI95%)	30 % (22.53 -- 38.72 %)	33.33 % (21.68 -- 47.46 %)	27.78 % (18.76 -- 39.05 %)
Indeterminant findings (Observer 2)	Absent	% (CI95%)	15 % (9.7 -- 22.47 %)	20.83 % (11.73 -- 34.26 %)	11.11 % (5.74 -- 20.42 %)
Indeterminant findings (Observer 2)	Flattening	% (CI95%)	85 % (77.53 -- 90.3 %)	79.17 % (65.74 -- 88.27 %)	88.89 % (79.58 -- 94.26 %)
Indeterminant findings (Observer 2)	Cortical sclerosis	% (CI95%)	0 % (0 -- 3.1 %)	0 % (0 -- 7.41 %)	0 % (6.94e-16 -- 5.07 %)

N: sample size; CI: confidence interval

## Data Availability

The datasets generated and/or analyzed during the current study are available from the corresponding author upon reasonable request.
